# Determining the length of disability and the severity of injury in patients with traffic accidents admitted to Imam Khomeini University Hospital in Urmia in 2016 

**Published:** 2019-08

**Authors:** Omid Garkaz, Shakar Salari Lak, Hamid Reza Mehryar, Hamid Reza Khalkhali

**Affiliations:** ^*a*^Urmia University of Medical Sciences, Urmia, Iran.; ^*b*^Department of Public Health, Islamic Azad University, Tabriz Branch, Tabriz, Iran.

## Abstract

**Background:**

Traffic accidents are recognized as the main cause of mortality and morbidity worldwide particularly in developing countries and it causes major health problems that endanger human health. This study aims to determine the disability period and the severity of traumatic injuries caused from traffic accidents. Patients refereed to Imam Khomeini University Hospital in Urmia during year 2016 were enrolled.

**Methods:**

This cross-sectional study was carried out on 1705 traffic accident victims which referred to Imam Khomeini University Hospital in Urmia during 2016. The data were collected using a checklist containing demographic characteristics, type of accident, and severity of injury and finally the length of disability period. Length of disability achieved by following up through telephone call. To determine the ISS score, according to the expert’s opinion, ranked 1-6 and evaluated.

**Results:**

In this study 1231 (72.1%) patients were enrolled. The average age of the patients was 33.63 (30.00) Seventy seven (0.04%) had fatal injuries, 593 (42.5%) of them returned to their normal activity after two months. The mean and standard deviation of return to normal was 86/78 (104/224). The majority of injured patients had a mild limb injury, while most injuries were life-threatening.

**Conclusions:**

In this study, most of the injured were male, married, young and concerned about compensation for financial losses, and most of the fatal injuries had a critical health hazard, which generally required crucial steps to improve health for quick return to work and reduce harm.

**Keywords:**

Injuries, Disability, Accidents

